# **Predictors of trips to food destinations**

**DOI:** 10.1186/1479-5868-9-58

**Published:** 2012-05-20

**Authors:** Jacqueline Kerr, Lawrence Frank, James F Sallis, Brian Saelens, Karen Glanz, Jim Chapman

**Affiliations:** 1 University of California, San Diego, USA; 2 University of British Columbia, Vancouver, Canada; 3 San Diego State University and University of California, San Diego, USA; 4 Seattle Children’s Research Institute and University of Washington, Washington, USA; 5 University of Pennsylvania, Pennsylvania, USA; 6 Urban Design for Health, Washington, USA

**Keywords:** Built environment, Food environment, Urban form, Travel, Nutrition, Obesity

## Abstract

**Background:**

Food environment studies have focused on ethnic and income disparities in food access. Few studies have investigated distance travelled for food and did not aim to inform the geographic scales at which to study the relationship between food environments and obesity. Further, studies have not considered neighborhood design as a predictor of food purchasing behavior.

**Methods:**

Atlanta residents (N = 4800) who completed a travel diary and reported purchasing or consuming food at one of five food locations were included in the analyses. A total of 11,995 food-related trips were reported. Using mixed modeling to adjust for clustering of trips by participants and households, person-level variables (e.g. demographics), neighborhood-level urban form measures, created in GIS, and trip characteristics (e.g. time of day, origin and destination) were investigated as correlates of distance travelled for food and frequency of grocery store and fast food outlet trips.

**Results:**

Mean travel distance for food ranged from 4.5 miles for coffee shops to 6.3 miles for superstores. Type of store, urban form, type of tour, day of the week and ethnicity were all significantly related to distance travelled for food. Origin and destination environment, type of tour, day of week, age, gender, income, ethnicity, vehicle access and obesity status were all significantly related to visiting a grocery store. Home neighborhood environment, day of week, type of tour, gender, income, education level, age, and obesity status were all significantly related to likelihood of visiting a fastfood outlet.

**Conclusions:**

The present study demonstrated that people travel sizeable distances for food and this distance is related to urban. Results suggest that researchers need to employ different methods to characterize food environments than have been used to assess urban form in studies of physical activity. Food is most often purchased while traveling from locations other than home, so future studies should assess the food environment around work, school or other frequently visited destinations, as well as along frequently traveled routes.

## Introduction

Some studies document built environment-obesity associations [1]‒[5]. Both physical activity environments and food environments could contribute to the relationship between obesity and urban form. It is well documented that neighborhood form (e.g., land use patterns) is related to physical activity [6]‒[9], but evidence regarding the relation of food environments to food purchasing patterns and eating behaviors is limited [10,11]. In the US, the number and distance to healthful food stores and restaurants varies by neighborhood income and ethnic composition [12]‒[15]. Indeed, most food environment studies have focused on income and ethnic disparities in obesity rates and diet quality and their relationship to availability of fast food restaurants and grocery stores [16]‒[18]. However, most studies of food environments in the U.S. have not considered urban form or other factors that impact access to food stores.

Definitions of food environment access and availability have included the number or density of food outlets in a given area and/or home-to-food outlet distances [4]. The often-applied gravity model asserts that closer destinations are exponentially more attractive, saving time and money on travel [19]. Trip tour data, however, also indicate that people piece together trips and stops to be convenient. Food outlet proximity is impacted by land use mix and street network patterns, with more gridded streets and a mixture of retail and residential land uses supporting shorter trips and more travel by walking and cycling [6]‒[8,20]. It is plausible that increased access to food (and other destinations) near where someone lives may result in less food purchasing to and from work and other destinations. One study showed that increased land use mix where people live resulted in simpler tours (less stops) to and from work [21]. Research has not yet examined if access to and use of healthy food stores is greater in these more ‘walkable’ neighborhoods [10], although one study found distance from home to food stores decreased with increasing population density, a marker of greater walkability [22]. The actual distance people go for food purchasing and the trip characteristics are understudied.

To date, environmental correlates of physical activity and obesity have been examined at scales up to a one mile buffer around residents’ homes [23,24], or even shorter distances for children [25,26]. These distances are reasonable estimates of how far individuals will walk, with the focus almost exclusively on environments around the home, where it is assumed most physical activity occurs. Walkability of neighborhoods measured at these scales is consistently related to walking for transportation in adults [6]‒[9]. In contrast, food environment studies mostly assess communities [27] or neighborhoods, defined by census blocks or tracts [28]. However, it is not clear what are meaningful scales or distances for defining food environments and/or whether food outlet type is important to scale/distance considerations. Obesity rates may also be impacted by the type of food outlet (store type). For example, grocery stores tend to sell higher quality and cheaper fresh fruits and vegetables [29,30], more low fat products and fresh products than fast food restaurants or convenience stores which tend to sell processed foods commonly high in fat and sodium [29,31].

The current study seeks to inform the understanding of the scale at which food purchasing from stores and restaurants should be evaluated by documenting, then examining correlates of how far people actually travel for food. The geographic scale at which food is obtained is likely a function of many factors including daily travel and commute patterns, presence of food options that match individual preferences and other individual factors (e.g., age), land use around the residence, income, and price. There is limited evidence on how far individuals actually travel for food [32] but getting food is the second most common travel purpose. Travel patterns for food are not well understood and it is not clear what proportion of food purchases are performed with home as the starting point. Associations of the food environment with diet and obesity could be obscured and results may be misleading if we continue to assume that food purchasing occurs only near one’s residence, and/or to use the same buffer sizes to measure the food environment as employed for physical activity environment studies. The association between residential neighborhood and obesity may be particularly misleading for low income ethnic groups most at risk for obesity, because these individuals spend large amounts of time away from their home neighborhood attending to family and work responsibilities [33]. Previous studies of food environments have not provided data on actual food purchasing behavior, where and when people buy food, and how far they travel to buy food in a large sample with trips extending beyond the local neighborhood.

## Methods

Data collected for the cross-sectional SMARTRAQ (Strategies for Metropolitan Atlanta’s Regional Transportation and Air Quality; see http://www.act-trans.ubc.ca/smartraq) household travel survey in the 13-county Atlanta region in 2001–2002 were analyzed. Data collection was stratified across 4 ranges of income and household size and 5 levels of residential density, meaning some population groups were oversampled to ensure variation in socio-demographics and urban form. Study method details are published elsewhere [5,23]. The overall response rate was typical for travel surveys at 30.4%; partly reflecting substantial study demands on participants. Verbal consent was acquired from participants and the study was approved by the local ethical review board. While the primary aim of the study was to study travel behaviors to inform transportation and air quality research and planning, the data included trips for the recorded purpose of eating or purchasing food which allowed the current analyses to be performed.

### Measures

The aim of this study was to explore environmental, individual and trip level factors related to shopping for food. The analyses were framed by the ecological model of behavior change that includes multiple levels of influence and would include factors from multiple types of environments e.g. home, work, school etc. Unfortunately in practice, most ecological studies to date focus only on the home environment. This study collected information from each location that was visited to allow a more complete analysis of travel for food predictors.

Participants completed a paper travel diary, recording destinations visited, travel mode and purpose, and time of day across two days assigned by the research team to ensure an even distribution of all weekday and weekend days across the sample. Socio-demographic information was provided by a head of household in a recruitment call through the use of a computer aided telephone interview (CATI) protocol. Height and weight were reported individually by household members. BMI was computed as kg/m^2^.

The built environment variables were created using Network Analyst, which is an extension to the GIS software product developed by the ESRI corporation known as ARCVIEW (ArcView GIS 3.2; ESRI Inc., Redlands CA, 2000). GIS was used to assess the distance participants traveled to food sources provided on the travel diary and to define the urban form around each address.

This study aimed to assess whether urban form variables related to physical activity and obesity were also related to food purchasing. A one kilometer road network buffer was developed around each trip origin, destination and home address to create urban form measures. A combination of county level Tax Assessors parcel data and census data were used to measure residential density and mixing of land uses, and street network files were used to measure street connectivity within the 1 kilometer buffer. These values were normalized for the sample and summed to create a measure of destination accessibility that has been related to both walking and vehicle miles travelled [21]. An index was employed to simplify the analyses because multiple locations were being investigated (origins, destinations and home) and comparisons across them could be made. These are the same methods published in two papers linking built environment measures with physical activity and obesity in adults from SMARTRAQ [5,23].

Car travel time and distance walked were calculated using GIS, with each trip, origin and destination from the travel diary placed on the street network. The shortest time (for car travel) and distance (for walking travel) between the origin and destination along the road network was computed. For car travel, expected travel times were developed based on time of day and direction of travel to adjust for congestion level, using data from the Atlanta Regional Commission’s Regional Travel Model. Corresponding morning, afternoon, and off peak zone-to-zone link-based travel times for reported trips were drawn from the regional travel model. The distances along the shortest route were then measured and employed in the current analyses.

### Identifying food location type

From a list, participants indicated the primary and up to four secondary activities they did at each destination and the destination name and address. Destinations were coded as food-related if one of the two food-related activities (purchasing food or eating) was recorded among the primary or secondary activities. Destinations only in which individuals indicated engaging in a food-related activity were categorized into food establishment types based on location name.

Food related activity destinations with food-related activities included convenience stores, bars, schools, churches, hospitals, entertainment centers and malls. Most of these were excluded from analyses as there were few trips to these destinations. Convenience stores were often visited but it was not clear that food shopping, as opposed to shopping for gas, had occurred. Many convenience stores are attached to gas stations, and there were few instances of eating reported in these locations. Trips to five food outlets were evaluated including to *fast food restaurants, sit down restaurants, grocery stores, coffee shops* and *large superstores*.

Destinations were assigned to the “fast food” category if they contained any of the following words: “burger, burrito, cafeteria, chicken, deli, food court, hot dog, pizza, sub, taco, wings”. Regional and national chain names, e.g. Burger King, Kentucky Fried Chicken, Krystal, McDonald’s and Mrs. Winner’s, were also included in the “fast food” category. Locations with the word “restaurant” in the name were included in the sit down restaurant group. After categorizing records into the above categories the remaining set of locations were reviewed for possible inclusion in the “restaurant” category. Locations were labeled sit-down restaurants based on a record-by-record review using known local/regional/nation restaurant chain names (e.g. Flying Biscuit, Fuddruckers, and Hard Rock Café), investigators’ knowledge of the region, and internet searches for location descriptions. Locations with the word “grocery” in the name were reviewed and included in the grocery stores category. Regional and national supermarket chains (e.g. Kroger, Publix), were also identified and included as ‘grocery stores’. The coffee shop category was developed from locations categorized by the research team as “bakery, doughnut shop, coffee shop, etc.” or if the location name included any of these words; “bakery”, “doughnut,” “bagel,” “bread” or “coffee.” Locations were categorized as “large superstores” if they were named one of the following—Belk Department Store, Costco Warehouse, Home Depot, Goody’s Family Clothing, Home Depot Expo, JC Penney, K-Mart, Kohl’s, Lowes, Rich’s, Sam’s Club, Sears, Target or Walmart. Although some of these stores are not principally food outlets, they were included if food eating/purchasing was reported by participants in these stores.

Participants who completed the travel diary and indicated at least one food related activity in either day at one of the 5 food establishment categories described above were included in analyses.

### Variables included in the analyses

For these analyses only food destinations were considered, and the immediate location before the food destination was considered the trip origin. The distance from the origin location to the food outlet was examined. The return trip was not included. For each trip origin and destination location, destination accessiblity scores were calculated from the index combining land use mix, intersection density and residential density. The urban form scores were split into tertiles based on these analyses.

For these exploratory analyses simple trips were considered in a set of predictive models. Travel behavior research generally considers more complex tours and trip chains [34,35]; often involving stops between home, work or other major non-work locations. To investigate the relationship between home residential environment and food purchasing a simple home-food-home tour category was created. This category included only trips from home to a food destination followed by a return trip to home, without other stops. Similarly, a simple work-food-work tour category was created. This category includes only trips from work to a food destination followed by a return trip to work, without other stops. Two other simple tours were created: a home-food-work tour and a work-food-home tour. The analyses investigated whether the travel behavior varied depending on whether these types of tours occurred.

Trip, personal and household variables were included as correlates of distance traveled for food purchasing. Distance and frequency of visits to the five food destinations were compared by day of the week, origin of the trip (home, not home) and urban form of the trip origin and destination. Household level income (<$50,000, $50-74,000, $75,000+) and number of vehicles owned were compared for distance and frequency of travel. At the person level, gender, race (white/non white), education (college degree or not), work status and obesity status (BMI greater than or equal to 30 or less than 30) were related to the dependent variables distance and frequency of travel.

### Analyses

Three dependent variables were analyzed; 1) Distance travelled for food (in miles, continuous), 2) Visit to a fastfood restaurant (vs visit to any other food location) and 3) Visit to a grocery store (vs visit to any other food location). Mixed model analyses were employed adjusting for clustering of trips by participant and household.

#### Environment factors

Tertiles of destination accessiblity in the one kilometer network buffer around the origin and destination of the food trip, and participants’ home was used.

#### Trip factors

These included day of the week (working day or not), whether the food trip started and ended at home, whether the food trip started and ended at work, the five categories of food locations (in the distance model only).

#### Participant demographic factors

Vehicles in the home, annual household income categories, educational status, employment status, obesity status, race, and gender.

## Results

A total of 116,541 trips were made by 7665 participants. Of these, 4800 participants made 11,995 trips that included a food activity (e.g., purchasing or eating food) during a visit to one of the five types of stores identified for these analyses. Across the two day diary period 31.1% of food trips were made to a grocery store, 29.9% to a sit down restaurant, 19.2% to a fastfood outlet, 13.1% to a superstore and 6.7% to a coffee shop which included a food purchase. Only 7% of all trips to a food outlet were made on foot.

### Distance traveled to any food store

The unadjusted mean distance travelled to each of the five food locations (and standard deviations) and for each independent variable can be found in Table 1. Table 1 also presents the results of the mixed methods modeling adjusting for person sampled and number of participants in the household. The data represent the trips made over the two day travel diary period.

**Table 1:** Distance to food stores

	**Unadjusted mean distance (SD) in miles**		*Adjusted T statistic	**P value**
**Outlet type visited on trip**				
Grocery store	4.67	(4.35)	−9.86	.001
Sit down	6.10	(4.87)	-.31	.75
Fastfood	4.96	(4.41)	−6.44	.001
Coffee shop	4.50	(4.40)	−7.02	.001
Superstore	6.32	(4.77)	Ref	
**Destination accessibility of trip origin location**				
Low	6.65	(4.51)	9.22	.001
Medium	4.99	(4.59)	1.39	.16
High	4.46	(4.57)	Ref	
**Destination accessibility of trip destination location**				
Low	6.92	(4.85)	9.23	.001
Medium	5.41	(4.59)	3.50	.001
High	4.56	(4.44)	Ref	
**Destination accessibility of home location**				
Low	6.40	(4.73)	5.54	.001
Medium	5.32	(4.47)	3.19	.001
High	4.47	(4.40)	Ref	
**Home food home tour**				
Trip not part of tour	5.34	(4.70)	1.32	.19
Home food home tour	5.37	(4.44)	Ref	
**Work food work tour**				
Trip not part of tour	5.42	(4.66)	3.67	.001
Work food work tour	4.38	(4.03)	Ref	
**Home food work tour**				
Trip not part of tour	5.32	(4.59)	−5.67	.001
Home food work tour	6.07	(5.38)	Ref	
**Work food home tour**				
Trip not part of tour	5.23	(4.54)	−9.91	.001
Work food home tour	6.51	(5.30)	Ref	
**Day of the week of trip**				
Non work day	5.36	(4.51)	2.33	.02
Workday	5.34	(4.73)	Ref	
**Household income level of trip participant**				
Income < $50,000	5.31	(4.73)	2.11	.04
Income $50,000–74,000	5.39	(4.50)	.54	.59
Income $75,000 +	5.34	(4.65)	Ref	
**Vehicle ownership in household of trip participant**				
No vehicles	4.76	(5.56)	−1.11	.27
At least 1 vehicle	5.36	(4.62)	Ref	
**Gender of trip participant**				
Male	5.36	(4.63)	.82	.42
Female	5.34	(4.63)	Ref	
**Education of trip participant**				
No degree	5.70	2.33	.02	
Degree	5.12	Ref		
**Ethnicity of trip participant**				
Non white	5.57	(4.78)	3.50	.001
White	5.30	(4.60)	Ref	
**Employment status of trip participant**				
Does not work	5.31	(4.48)	−1.61	.11
Works	5.34	(4.73)	Ref	
**Age (continuous)**	-	−1.49	.14	

* adjusted for all independent variables as well as person taking the trip and number of people in household taking trips.

Participants travelled furthest for superstore food shopping and the least distance to grocery stores and coffee shops. Those living in less accessible environments or making trips to and from less accessible environments traveled farther.

Participants travelled further to food stores when the trip was part of a larger tour with differing origins and destinations before and after the trip to the food location; i.e., work food home tour or home food work tour. When the tour was work food work, distances travelled were shorter. Participants travelled farther on non-work days for food.

In the adjusted analyses, lowest income participants, non whites, and those without a degree travelled further for food.

### Grocery stores

Table 2 presents the percentage of trips made to the grocery store by environment, trip and person level variables and the results of the adjusted analyses. Those starting a trip from the least accessible neighborhood were less likely to visit a grocery store than those starting from the most accessible. Those travelling to a highly accessible destination were less likely to visit a grocery store than those traveling to a medium accessible community. The destination accessibility of the home environment was not significant.

**Table 2:** Grocery store visit

	**Unadjusted%**	*Adjusted T statistic	**P value**
**Destination accessibility of trip origin location**			
Low	28.1	−3.02	.003
Medium	34.3	.79	.43
High	31.9	Ref	
**Destination accessibility of trip destination location**			
Low	29.4	1.65	.10
Medium	34.4	4.28	.001
High	29.5	Ref	
**Destination accessibility of home location**			
Low	28.0	−1.60	.11
Medium	32.4	.39	.70
High	32.7	Ref	
**Home food home tour**			
Trip not part of tour	28.9	−7.47	.001
Home food home tour	37.7	Ref	
**Work food work tour**			
Trip not part of tour	32.9	9.73	.001
Work food work tour	5.9	Ref	
**Home food work tour**			
Trip not part of tour	31.8	4.78	.001
Home food work tour	15.9	Ref	
**Work food home tour**			
Trip not part of tour	29.5	−9.57	.001
Work food home tour	44.9	Ref	
**Day of the week of trip**			
Non work day	36.1	2.01	.05
Workday	27.0	Ref	
**Household income level of trip participant**			
Income < $50,000	35.6	3.53	.001
Income $50,000-74,000	30.9	2.06	.04
Income $75,000 +	27.6	Ref	
**Vehicle ownership in household of trip participant**			
No vehicles	43.3	2.02	.04
At least 1 vehicle	30.9	Ref	
**Gender of trip participant**			
Male	26.6	−5.70	.001
Female	34.8	Ref	
**Education of trip participant**			
No degree	31.9	-.46	.65
Degree	30.5	Ref	
**Ethnicity of trip participant**			
Non white	36.8	5.79	.001
White	29.7	Ref	
**Employment status of trip participant**			
Does not work	37.6	.35	.73
Works	28.7	Ref	
**Obesity status (from BMI) of trip participant**			
Not obese	32.1	−2.63	.009
Obese (BMI 30+)	29.7	Ref	
**Age (continuous)**	6.74	.001	

* adjusted for all independent variables as well as person taking the trip and number of people in household taking trips.

People were more likely to travel to a grocery store with a tour starting and ending at home and less likely to travel to a grocery store during a break from work (i.e. a work food work tour). Participants were less likely to visit a grocery store on the way to work and more likely to visit a grocery store on the way home from work. Participants, however, were most likely to visit a grocery store on a non work day.

Those in high income households were least likely to visit a grocery store compared to low and mid income groups over the two day survey period. Those without a car were more likely to visit a grocery store than other types of food store. Men were less likely to visit a grocery store than women. Relative to their counterparts, non whites, non-obese participants and older adults were more likely to visit a grocery store.

### Fast food outlets

Table 3 presents the results of the unadjusted and adjusted analyses for the dependent variable, visit to a fastfood outlet.

**Table 3:** Fastfood outlet visit

	**Unadjusted%**	*Adjusted T statistic	**P value**
**Destination accessibility of trip origin location**			
Low	18.3	-.60	.55
Medium	18.4	−1.78	.08
High	19.8	Ref	
**Destination accessibility of trip destination location**			
Low	18.5	−1.11	.27
Medium	19.1	-.23	.82
High	18.8	Ref	
**Destination accessibility of home location**			
Low	20.1	2.39	.02
Medium	19.0	1.54	.12
High	18.5	Ref	
**Home food home tour**			
Trip not part of tour	21.2	7.13	.001
Home food home tour	13.0	Ref	
**Work food work tour**			
Trip not part of tour	18.0	−7.51	.001
Work food work tour	34.9	Ref	
**Home food work tour**			
Trip not part of tour	18.9	−1.93	.05
Home food work tour	25.0	Ref	
**Work food home tour**			
Trip not part of tour	19.8	5.68	.001
Work food home tour	13.9	Ref	
**Day of the week of trip**			
Non work day	16.1	−2.57	.01
Workday	21.7	Ref	
**Household income level of trip participant**			
Income < $50,000	20.7	3.51	.001
Income $50,000-74,000	18.8	1.53	.13
Income $75,000 +	18.2	Ref	
**Vehicle ownership in household of trip participant**			
No vehicles	19.8	.80	.42
At least 1 vehicle	19.1	Ref	
**Gender of trip participant**			
Male	20.5	2.75	.006
Female	18.0	Ref	
**Education of trip participant**			
No degree	21.1	2.37	.02
Degree	17.7	Ref	
**Ethnicity of trip participant**			
Non white	19.6	−1.28	.20
White	18.9	Ref	
**Employment status of trip participant**			
Does not work	16.2	1.79	.07
Works	19.4	Ref	
**Obesity status (from BMI) of trip participant**			
Not obese	17.9	−2.74	.006
Obese (BMI 30+)	21.7	Ref	
**Age (continuous)**	-	−5.50	.001

* adjusted for all independent variables as well as person taking the trip and number of people in household taking trips.

The accessibility level of the trip origin and destination was not related to visiting a fast food outlet. Trips to fastfood were more likely when the home neighborhood environment was least accessible vs most accessible.

Trips to fastfood were more likely to made on work days than non work days. Tours to and from home were less likely to result in a fastfood stop. Tours that started and ended at work were more likely to include a visit to a fastfood restaurant. Tours from home on the way to work were more likely to include a fastfood stop. Tours from work on the way home were less likely to result in a fastfood stop.

Men were more likely to visit a fastfood outlet that women. Those earning less than $50K were more likely to eat fastfood than those earning $75K or more. Those without a college degree were more likely to visit a fastfood outlet. Older participants were less likely to visit a fastfood outlet. Obese adults were more likely to visit a fastfood restaurant.

## Discussion

In this study of Atlantans, the lowest average distance traveled to a food establishment was greater than 4.5 miles. This is a much longer distance than the 1-km to 1-mile buffers around homes that are often constructed in studies of physical activity environments [23]. Therefore we have to be cautious about interpreting the relationship between obesity if it is assumed that an individual’s food environment is constituted only or mostly within these buffers.

Although many food trips are currently outside of the residential neighborhood, a 1-km buffer is still a good indicator of food access that may be related to shorter journeys, more walking for transportation and greater support of local food sources. Participants travelled furthest to larger superstores for food, as might be expected based on variety and cost considerations. The implications for greenhouse gas emissions and air pollution for longer trips to superstores should be evaluated relative to potential efficiencies if fewer trips to obtain food overall are made. Travel for food or *food miles* is becoming a central focus of strategies to reduce greenhouse gas emissions [36].

Food trips were longer when people started from a non-home location or lived in suburban type location without connected streets, mixed land uses or high residential density. Non-white, those without a degree, and lower-income subgroups travelled longer distances for food, suggesting local food sources may be unavailable and travel to food may be an additional hardship for the underserved. Increased time spent in cars may also be related to obesity [23]. In one study, supermarkets were on average 1.15 miles further away for residents of black compared with white neighborhoods [37]. In another study, researchers estimated that residents of low-income neighborhoods would have to travel more than 2 miles to have access to the same number of supermarkets as residents of higher-income areas [32]. Our findings for actual travel distances for food indicated that local access to foods around the home is less frequent than access to foods along routes that occur as part of people’s everyday lives. This may be the result of home environments devoid of local food outlets. Examining accessibility along common routes requires a completely different measurement strategy than focusing only on residential neighborhoods and could include use of GPS devices which track individuals across multiple locations and routes [38]. This is consistent with current tour based approaches to modeling and predicting travel behavior where food outlet visits are often a mid tour stop [20].

Origin and destination environment, type of tour, day of week, age, gender, income, ethnicity, vehicle access and obesity status were all related to visiting a grocery store. Home environment, day of week, type of tour, gender, income, education level, age, and obesity status were all related to likelihood of visiting a fastfood outlet. Many of the same results were seen for the grocery store but operating in the opposite direction. For example men were more likely to visit a fastfood outlet and less likely to visit a grocery store, and older adults were more likely to visit a grocery store than a fastfood location. Fastfood outlet visitation (and dining out overall) may represent a less healthy lifestyle with mostly high fat processed foods available, and grocery store visits may represent a healthier lifestyle because fresh fruits and vegetables are available.

More trips were taken to fastfood on working days than non working days (and vice versa for grocery stores). In particular, a break during the work day was likely to include a fastfood trip, as was the journey to work at the start of the day. This suggests that when participants are pressed for time they resort to stopping for fastfood. Interventions could target these particular trip habits and suggest preparing a healthy breakfast and lunch at home and taking it to work to avoid such unhealthy stops or alternately the provision of healthful food options by employers.

While it is now possible to order healthier options at fastfood outlets, the relationship between obesity and fastfood outlet visitation suggests that patrons are still purchasing high fat foods. Continuing to work on programs to provide healthier options where stops are made is important. One study found that visiting a fast food restaurant was a significant predictor of higher BMI and visiting a grocery store a significant predictor of lower BMI in women [39].

Those starting a trip in a neighborhood with few destinations were less likely to travel to a grocery store, but those traveling to a medium accessible environment were more likely to visit a grocery store. This may reflect the availability of parking at many grocery stores, which although surrounded by other destinations, would contribute to the grocery store being in a moderately accessible community. Some unexpected results were found for grocery store visits, including low income participants, non whites and those without a vehicle visiting a grocery store more often than other types of stores. Perhaps those without a car cannot carry as much food with them in a single journey and are more likely to make smaller, frequent trips to the grocery store.

The present study clearly shows that people get food from a variety of locations, many of which are outside of their local community. Therefore, examining the residential food environment alone is insufficient. Many trips to purchase food begin at locations other than home. This suggests studying locations that individuals frequent may be more useful than estimating access to food only around the home; many people spend as much or even more of their waking hours at work. One study found that fast foods restaurants tended to cluster around schools [40]. To date, few studies have investigated whether the availability of healthy or unhealthy foods outside of the residential environment, along the routes or ‘activity space’ of an individual, is related to diet or obesity [38]. One small study of migrants did collect a travel and food diary over a week period to assess access to foods locally. [41]. A recent review also emphasizes the need to study locations other than home neighborhoods and food environments associated with commuting behaviors [42]. Fruitful locations may be around workplaces or along frequently traveled routes.

Although the literature has documented some disparities in obesity related to differences in healthy food access (6 out of 10 studies) [43], these studies have not directly assessed where food was purchased. Our study indicated that income, ethnicity, and education was related to distance travelled. Studies suggests that food deserts may be racially driven, not just income related [44]. Another explanation comes from studies that have also shown that some population groups spend large amounts of time traveling outside of their home environments due to work and care commitments [23,33]. Assessing food purchasing patterns outside of home neighborhoods may be particularly important for this group.

The strengths of this study were the large and diverse sample as well as specific measures of places where individuals purchased food. The limitations included use of an activity-based travel survey that relied upon self-report of trips and activities, and limiting the environment to an urban/suburban sample. One study indicated that distances to food stores may be even greater in rural areas [22]. A two-day travel diary may not be representative or inclusive of the habitual food outlet visitation for an individual or a household. The response rate was lower than for a typical survey study, but the respondent burden for the present study was higher, and there was no information collected about non-respondents. The study did not examine the possible food stores that were available to travel to (and/or their quality), making it difficult to determine whether respondents were making trade-offs regarding distance to/from food outlets and quality; i.e., traveling further to get better quality or cheaper food, or simply choosing to purchase food while on a trip. More complex food tours should be analyzed in future studies, and new techniques from transportation research may be enlightening, such as employing mental mapping of daily travel behavior [45]. Food trips were not compared to trips for other purposes.

Enumerating food locations accurately [46] and conducting quality audits [29] are both labor-intensive processes that would be prohibitive for the 13-county Atlanta region. This study would be strengthened by specific food intake data or food purchasing receipts to confirm what food locations were most impacting participants’ diet and weight. A recent study in the Waterloo Region of Ontario Canada known as “NEWPATH” was patterned after the Atlanta SMARTRAQ study but also included dietary data collection if a location was visited that involved a food purchase [47]. Exclusion of convenience stores due to the unclear activity criteria was a study weakness as these are common locations for food purchasing. Additional food locations should be considered beyond the five primary ones in the current analyses and inclusion of food locations where food can be grown not just purchased may be informative.

The present study demonstrated that people travelled sizeable distances for food and this distance is related with urban form. Results strongly suggest that researchers need to employ different methods to characterize food environments than have been used to operationalize built environment in studies of physical activity. Further, food is most often purchased while traveling from locations other than home, so future studies should assess the food environment around work, school or other frequently visited destinations, as well as along frequently traveled routes. Increasing our understanding of travel patterns to purchase food is important for improving our health and the health of our environment.

## Competing interest

The authors have no competing interests to declare.

## Authors’ contribution

JK analyzed the data, drafted and edited the manuscript. LF designed the study, collected the data, drafted and edited the manuscript. JS drafted and edited the manuscript. BS drafted and edited the manuscript. KG drafted and edited the manuscript. JC collected the data, created the variables and contributed sections to the manuscript. All authors read and approved the final manuscript.
